# Development and validation of a questionnaire about hidden curriculum in medical institutes: A pilot study

**DOI:** 10.3389/fmed.2023.996759

**Published:** 2023-02-09

**Authors:** Ayesha Rauf, Fozia Fatima, Rehama Gilani, Nadia Shabnam

**Affiliations:** ^1^Department of Health Professions Education, National University of Medical Sciences, Islamabad, Pakistan; ^2^Department of Public Health, National University of Medical Sciences, Islamabad, Pakistan

**Keywords:** hidden curriculum, exploratory factor analysis, psychometric properties, medical institutes, questionnaire, Pakistan

## Abstract

The goal of the current study was to develop and validate a questionnaire that would reveal characteristics of medical hidden curricula. It is an extension of the qualitative research that was done by researchers on hidden curriculum, and a second part of the qualitative was the creation of a questionnaire by a panel of experts. Using both exploratory factor analysis (EFA) and the quantitative portion, the questionnaire was verified. The sample size was 301, and the participants, who were from medical institutes, were both genders and between the ages of 18 and 25. First, a thematic analysis of the qualitative portion was used to create a 90-item questionnaire. The validity of the questionnaire’s content was certified by the expert panel. A 39-item questionnaire was subsequently created after the items that overlapped and the items that did not represent the particular theme were eliminated. After that, we validated the survey. A total of 39 high-loading components made up the six variables of EFA, which explained 62% of the variance. The 33-item questionnaire, from which six items were deleted, was found to have satisfactory psychometric qualities. As a result, the accountability of faculty and students in curricula and extracurricular activities combined with equal opportunity is one factor, communication and relationships with stakeholders combined with evidence-based reforms and implementations are the second factors, and student-centeredness and empowerment as the third main factor of the hidden curriculum are all considered to be important factors. All these three main constructs were collectively used to measure hidden curricula in medical institutes.

## Introduction

Recent curriculum changes in medical education and training have not resulted in a significant change (1). There are various reasons for this; one school of thought holds that schools ought to refocus on providing students with a more enabling and empowering learning environment rather than rigid learning and assessment of students (2). These contexts have a strong foundation in an alternate construct, where formal, informal, and hidden curricula, despite being connected, have distinct influences and independent roles. Although necessary for accomplishing effective educational objectives, a formal curriculum of a standard level is not the only factor that affects students’ progress and knowledge (3).

A barrier or facilitator can also be an environment where pupils learn, whether it be internal or external, material or intangible. As its essential components are not defined nor studied in a local context, there is a dearth of research regarding the influence of hidden curriculum practices on student learning. A general definition of the hidden curriculum is the attitudes and values that are frequently implicitly and tacitly, and perhaps accidentally, communicated through the educational structures, procedures, and culture of an educational institution. According to this definitional framework, researching medical education entails acknowledging the significance of context above all else. Every interaction, from the dyad to entire societies, takes place in the context of a variety of external elements, some of which have a significant impact on the scenario we are trying to comprehend. Even if most of what happens in organizational contexts is tacit, it is nonetheless widely accepted and communicated (4). Although the components of hidden curriculum have been elucidated in previous studies (5) and to some extent in this work, it may be stated that the majority of recognized components can be handled. The scope of the so-called “hidden curriculum” in medical education has grown steadily since it was first discussed in the 1960s and applied to medical education in 1994.

The phrase was first used in reference to medical education by Frederic Hafferty as the “collection of influences that function at the level of organisational structure and culture.” In general, he defined the hidden curriculum as “‘understandings,’ customs, rituals, and taken-for-granted aspects of what goes on in the life-space we call medical education,” and he believed that the hidden curriculum in medical education is an institutional-level concept that is best visible in “(1) policy development, (2) evaluation, (3) resource allocation, and (4) institutional slang” (6). A variety of “hidden” aspects of teaching and learning have been revealed and explained by scholars working in all areas of medical education over the past 20 years. In fact, in many ways, the impacts of what is referred to as the hidden curriculum are more pervasive than those of the formal curriculum. Their effects are rarely benign (7). Therefore, there is no need to worry about the unforeseen and negative effects the disguised curriculum may have. Instead, it can be utilized as a tool to assist graduates in acquiring the qualities and skills they desire (8). This research is an extension of earlier efforts by scholars to identify several components of covert curricula at Pakistani medical colleges. In order to assess important components of concealed curricula, we want to create a questionnaire with a local context. The goal of our study is to analyze the major methodological issues in the development of the questionnaire and measure the reliability and validity of the questionnaire by exploratory factor analytic techniques because the interpretation of such results depends on instrument reliability and validity (9).

## Literature review

Studies on educational environments are where the idea of the hidden curriculum in literature originates. In the 1960s and the first part of the 1970s, a critical mass of work on the HC started to form. The HC concept is used in many different educational contexts nowadays. Research using the HC as an analytical lens is still more prevalent in sociology than in education. The three terms “social systems,” “social structure,” and “organizational culture” are used to describe the emphasis sociologists place on implicit influences (10). Thus, it follows that the intellectual roots of the hidden curriculum (HC) can be found in the disciplines of sociology and education (8). Sociologists started paying critical attention to how social institutions affect individual behavior in the later half of the 19th century, as well as to the overt and covert subjective meanings people give to their own and other people’s social behavior. Later, social scientists started to investigate how certain actions came to be accepted as normal, examining the relative influence of formal vs. informal social norms and the crucial part that social relationships played in the development of group norms and culture. These research areas sparked study in the 1940s and 1950s on the formal and informal organization of professional groups like medicine, particularly the need to distinguish between the curriculum on paper and what academics were starting to call the “informal curriculum.” But before the phrase “hidden curriculum” would first exist, it would be long into the 1960s (10).

Two of the early studies of medical education, The Student-Physician by Robert K. Merton and colleagues (1957) and Boys in White by Howard Becker and colleagues (1961), had a profound impact on what would later be referred to as “the hidden curriculum.” Merton’s studies on the distinction between apparent (conscious and deliberate) and latent (unconscious and inadvertent) functions, his theory of unintended consequences, and his work on role modeling and modeling served as the direct inspiration for the work on the hidden curriculum. Given Becker’s extensive earlier research on educational settings and the effects of social stratification, social class, and social status on student learning, as well as his later scholarship on latent culture, latent social roles, and the existence of a “student culture” in medical schools, the connections are more obvious. Not to mention, “location” had a vital role in the conceptual tool development of the HC (10).

In the first half of the 20th century, studies on the subject of education started to emerge with a focus on separating the formal and informal aspects of educational practices. In order to highlight the variety of learning contexts that exist outside of the classroom, John Dewey coined the term “collateral learning,” which he used to characterize learning that occurs while doing other tasks (11, 12). Meanwhile, other people started mentioning “the experience of the curriculum in action,” “the informal learning experiences in the school,” and “the impact of informal education.” The idea that education is a “sociocultural process” one in which personality development and cultural reproduction coexist as interdependent forces arose. Due to the peculiarities of the hidden curriculum, it is necessary to draw pertinent conclusions from the conceptual framework. Different actors, including students, teachers, society, and variables like perceptions, attitudes, and awareness, call for conscious thought (13). Some elements operate as facilitators, allowing students to explore the learning paths by providing them with information about the hidden curriculum, for example. But those who are ignorant continue to be at a disadvantage, which leads to difficult circumstances (14).

The conventional educational system offers various opportunities that can either advance students’ intellectual development or hinder it. Individual learning and the goals of a formal curriculum may not always correlate linearly. It reacts to an environment that can provide information and cues that are necessary for the formation of attitudes, beliefs, and values (15). The unexpected, meaningful learning that occurs outside of the confines of the prescribed curriculum has a big impact on how students learn and grow (16). The phrase “hidden curriculum” (HC) refers to non-academic schooling results and by-products that have not been examined, and are covert, latent, unwritten, unplanned, and invisible (17). These phrases offer suggestions about several facets and fundamental ideas of HC. Even though learning through HC may or may not correspond to the formal curriculum’s goals, it can have positive effects. To prevent such learning, medical schools take extreme precautions. The hidden curriculum explains how kids learn by participating in rigorous planning, organization, and resource provision by the school while not physically participating in such activities or being taken into consideration by school authorities.

Many intangible characteristics such as knowledge, attitudes, emotion, cognition, intention, or behavior are measured by questionnaires as it tends to seize respondents’ perceptions through their self-reported observation (9). Study participants respond to a series of questions that are numerically coded and statistically analyzed. The success of the data collection tool depends upon the ability of items to measure what they are supposed to measure. Essentially, the questionnaire items must be able to reliably translate the key concept within the context of a research question and are generalizable to the target population (18). The advantage of questionnaires is that they are administratively convenient, economical, and statistically simpler to analyze (13). However, interpreting the item statement by respondents like researcher intent and understanding, even if both share the same language, is debatable and draws criticism (18). Restricting items to close-ended questions reduces the depth and richness of data (1). Therefore, using a questionnaire as a data collection tool in areas where little is known is not considered an appropriate methodology (9).

## Methodology

### Participants

A total number of 301 students of private and public medical colleges joined the study to provide their perceptions on the hidden curriculum. The students were randomly selected, based on a simple random sampling technique, from two major sectors of education, one private and the other public sector. There were 150 males (50%) and 151 females (50%), age groups ranging from 18 to 25 years old, and different academic years (1st–5th year). Participants’ native language was Urdu with English as a medium of instruction (see Table 1).

**TABLE 1:** Demographic information of the study participants.

Variables	Frequency (%)
**Sectors**	
Public	160 (53.0)
Private	141 (47.0)
**Gender**	
Male	150 (49.83)
Female	151 (50.0)
**Academic years**	
First year	50 (16.6)
Second year	76 (25.25)
Third year	40 (13.3)
Fourth year	63 (20.9)
Fifth year	72 (23.9)
**Region**	
Urban	180 (60.0)
Rural	121 (40.0)

### Research design

An exploratory sequential mixed methods design was used to conduct the study. A mixed-methods study design known as an exploratory sequential design places the quantitative part of data collecting and analysis after the qualitative phase. Its benefits include simplicity and opportunities for in-depth investigation of the quantitative outcomes (18). The design included 4 phases in total. The First 2 phases covered the questionnaire development and the remaining 2 phases covered the questionnaire validation.

#### Phase 1

**FIGURE 1:**
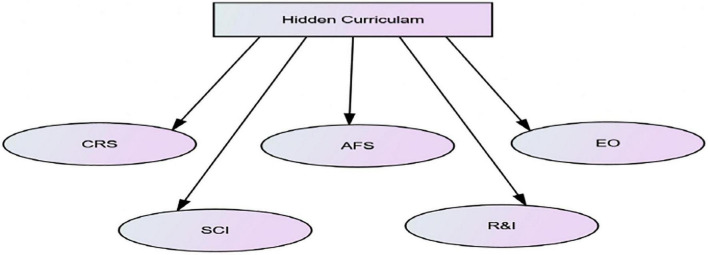
Dimensions of hidden curriculum. CR, *communication and relationship with stakeholders; AFS, accountability of faculty and students in curricular and extracurricular activities; EO, equal opportunity; SCE, student centredness and empowerment; and RI, Evidence based reforms and implementations.

It was an extension of the researchers’ qualitative study of Rauf et al. (1). The qualitative part’s extracted themes were used to conceptualize the constructs. Communication and relationships with stakeholders (CR), accountability of faculty, staff, and students (AFS) in all academic and extracurricular activities (AFS), morality in social interaction (SI), student-centeredness and empowerment (SCE), and academic challenges through evidence-based reforms and implementation (RI) (see Figure 1) were identified as the hidden curriculum’s key components from the qualitative portion of the study (1). These commonly occurring themes cut across all implicit activities of an institute and may have tangible and intangible implications on the immediate learning and future professional and personal life of the student.

#### Phase 2

This stage involved the creation of survey items and the modification of the questionnaire by expert panels to achieve sufficient content validity. The questionnaire was created using data from a thorough assessment of the literature, we not considered these items in-depth interviews (10 from private and public medical institutions, respectively), field observations, and document analysis. The expert panel featured professionals from a variety of fields, including those who specialize in education, public health, and the health professions. These specialists can help the study identify the questionnaire’s content validity more precisely. The minimum qualifications for selection of the specialists were a master’s degree or a doctorate and 10 years of professional experience. After the specialists in the field of health professions education examined the content validity, the original version’s 90 items were cut to 39 items. The questionnaire was revised before the collection of the final data in accordance with the advice of experts to ensure translational validity (face validity). The questionnaire was developed and designed in the English language as a medium of instruction in all medical colleges across Pakistan is English.

#### Phase 3

The drafted questionnaire was carried out on 301 students of two private and public medical schools in Rawalpindi. The study was approved by Institutional Review Board (IRB). Data were collected anonymously and participation was voluntary. The written questionnaire was provided to the participants electronically (google Docs form). The distribution and completion of the questionnaires took 3 months. The final version of the constructing and validating hidden curriculum scale in private and public medical schools for students consisted of a 39-item questionnaire which was grouped into six factors: Communication and relationship with stakeholders (CR) six items, Accountability of faculty and students in curricular and extracurricular activities (AFS) with eight items, Equal opportunity (EO) with eight items, Student centredness and empowerment (SCE) with nine items and Evidence-based reforms and implementations (RI) with eight items. The items were evaluated using a Likert scale with a scoring range between 1 (strongly disagree) and 5 (strongly agree).

#### Phase 4

The factor structure of the instrument was investigated and estimated using exploratory factor analysis. This phase’s primary goal was to determine the strength of the questionnaire’s intended items and reduce their quantity. To determine the conceptual direction of the construct, reverse scoring was used. Prior to EFA, Bartlett’s test of sphericity assumptions and the Kaiser–Meyer–Oikin (KMO) measure of sample adequacy were both put to the test. The Cronbach’s alpha coefficient was also used to examine the instrument’s internal consistency. All items’ descriptive statistics, including averages and standard deviations, as well as bivariate correlations, were also acquired. The statistical program SPSS 27.0 was used to analyze the data.

## Analysis and results

A sample of 301 was drawn by a convenient sampling method from the medical colleges of Rawalpindi and Islamabad. The participants of the research belong to different regions of Pakistan. The instrument consists of 5 constructs and 39 items. The analysis was disaggregated into two parts. In the first part, we explore the data and find the reliability of the scale and item-wise reliability. The results show that all the 39 items related to five constructs of hidden curriculum are reliable with a value of alpha (0.962). The results in Table 2 also indicate the item-wise reliability and all values are greater than 0.80.

**TABLE 2:** Items, alpha, mean, and standard deviation for the construct of hidden curriculum.

Items	Mean	SD	Alpha	Mean	SD
**Communication and relationship with stakeholders (CR)**					
CR1	3.62	0.838	0.840	3.680	0.696
CR2	3.70	0.885			
CR3	3.65	1.066			
CR4	3.90	0.856			
CR5	3.63	0.983			
CR6	3.58	0.954			
**Accountability of faculty and students in all academic and non-academic activities (AFS)**					
AFS1	3.87	0.875	0.833	3.591	1.030
AFS2	3.40	0.857			
AFS3	3.81	0.848			
AFS4	3.07	1.001			
AFS5	3.48	1.063			
AFS6	3.76	1.050			
AFS7	3.73	0.954			
AFS8	3.60	0.969			
**Equal opportunity (EO)**					
EO1	3.64	0.965	0.909	3.601	0.648
EO2	3.41	1.182			
EO3	3.80	0.907			
EO4	3.53	1.025			
EO5	3.43	1.174			
EO6	3.75	0.977			
EO7	3.64	0.950			
EO8	3.60	1.027			
**Student centeredness and empowerment (SCE)**					
SCE1	3.62	0.922	0.908	3.69	0.926
SCE2	3.54	1.044			
SCE3	3.54	0.953			
SCE4	3.61	0.908			
SCE5	3.79	0.855			
SCE6	3.96	0.844			
SCE7	3.97	0.832			
SCE8	3.54	0.991			
SCE9	3.66	0.975			
**Evidence-based reforms and implementation (IR)**					
RI1	3.62	0.835	0.825	3.46	1.044
RI2	3.50	0.961			
RI3	3.46	1.008			
RI4	3.37	1.001			
RI5	3.87	0.815			
RI6	3.73	0.863			
RI7	3.79	0.883			
RI8	2.39	1.706			

The mean and standard deviation of the first construct communication and relationship with stakeholders is $\left(\bar{x}=3.680\,a\,n\,d\,\sigma =0.696\right)$. The results show that among the items belonging to the communication and relationship with stakeholders: the vision of institutes is progressive have a higher area $\left(\bar{x}=3.90\right)$ as compared to the other items of the respective construct. The accountability of faculty and students in all academic and non-academic activities has an average value of 3.591 a and value of SD is 1.030. The item teachers attitude encourages students to ask questions has a higher average value σ is 0.875. If we look at equal opportunities of gender in medical colleges then it is observed that cultural diversity in our institutes is highly respected $\left(\bar{x}=3.80\&\sigma =0.907\right)$. Similarly, the role of institutions to help the students in their future and appreciation of academic achievement has greater importance the student-centeredness and empowerment. The reason for academic achievement depends on the evidence-based learning $\left(\bar{x}=3.73\right)$ and classroom practices by experienced and skilled faculty and also encourages the students’ research $\left(\bar{x}=3.79\right)$. The 39 items of the hidden curriculum were subjected to Exploratory Factor Analysis (EFA) using SPSS Version 27. Before carrying out EFA, the appropriateness of data for factor analysis was evaluated based on various criteria. The correlation matrix showed the presence of many coefficients of 0.3 and above. The Kaiser–Meyer–Oklin value was 0.87, greater than the recommended value of 0.6 and Bartlett’s Test of Sphericity reached statistical significance, assisting the factorability of the correlation matrix. EFA explored the presence of six components with eigenvalues exceeding one, explaining 44.16, 4.91, 4.23, 3.46, 2.82, and 2.69% of the variance, respectively.

Oblimin rotation was done in addition to the interpretation of these six components. The rotated solution revealed the existence of a simple structure, with all variables heavily loading on just one component and all six components displaying many strong loadings. The six components’ interpretation accorded with earlier studies on the hidden curriculum scale. Each item on the structure pattern was identified, labeled, and coded under the relevant theme of the questionnaire. Before factor analysis, the questionnaire contained the first theme “Communication and Relationship with stakeholders (CR)” had 06 items; Accountability of Faculty and Students in Curricular and Extracurricular activities (AFS) contained 08 items; Equal Opportunity (EO) contained 08 items; Student Centeredness and Empowerment (SCE) contained 09 items and Evidence-based reforms and Implementations (RI) contained 08 items. Items of the same theme were clubbed. Factors having the highest eigenvalue of items were identified, labeled, and coded. Themes loading separately or on the same factors were identified and including Accountability of Faculty and Students in Curricular and Extracurricular (AFS) plus Equal Opportunity (EO) were identified at factor 2. Similarly, Student Centeredness and Empowerment (SCE) were identified in factor 3; Communication and Relationship with stakeholders (CR) plus Evidence-based reforms and Implementations (RI) were identified in factor 5. Items that did not conform with the labeled constructs including AFS 2 and 4 and RI 6, 7, and 8, and Item 6 of SCE were deleted.

Exploratory factor analysis constituted six factors with 39 high-loading items, which extracted 62% of the variance. It was observed that the 33-item questionnaire (detached six items due to its outlier nature) was satisfied with good psychometric properties.

## Discussion

This study intended to develop a questionnaire that can evaluate hidden curricula in medical institutes. We followed the standard methodology of questionnaire development, item generation, scale development, and scale validation. We started the domain boundaries and created items centered on the constructs of the hidden curriculum. Firstly, we created a 90-item question pool. The expert panel ensured the content validity of the 39-item questionnaire, and pre-testing confirmed the cognitive debriefing for face validity. EFA removed the low-loading items and perceived severity items, providing the three-factor model with 33-item questionnaires. We dropped the severity items during EFA for many reasons. During analysis, one of the constructs has no chance to select. Secondly, while running step-by-step EFA for item refinement, these severity items were cross-loaded to more than one factor; hence we detached them. Thirdly, we removed some of these items while going through the process of internal consistency of constructs. This will increase the reliability of the constructs (18). These severity items could not strongly correlate to form a factor like other latent factors (see Tables 3–5). It is because the results of construct validity and internal consistency reliability help to determine which factors (items) should remain on the scale and how each factor on the test relates to each other. They are applied to groups of variables that are suggested to gauge several aspects of a single notion. Their procedures operate so that a single component only affects a single idea feature. The results of Cronbach’s alpha coefficient, item-total correlations, inter-item correlations, and Cronbach’s alpha if item deleted scores can be used to quantify them. However, the exploratory factor analysis-based instrument’s content validity, construct reliability, and discriminant validity will all be tested before the scale’s real validity is established (19).

**TABLE 3:** Total variance explained.

Items	Initial eigenvalues			Extraction sums of squared loadings			Rotation sums of squared loading
	Total	% of variance	Cumulative %	Total	% of variance	Cumulative %	Total
1	17.224	44.163	44.163	17.224	44.163	44.163	12.643
2	1.916	4.914	49.077	1.916	4.914	49.077	9.978
3	1.648	4.226	53.303	1.648	4.226	53.303	6.835
4	1.351	3.463	56.766	1.351	3.463	56.766	3.068
5	1.100	2.821	59.587	1.100	2.821	59.587	10.799
6	1.047	2.685	62.272	1.047	2.685	62.272	4.281
7	0.965	2.475	64.748				
8	0.886	2.273	67.021				
9	0.862	2.209	69.230				
10	0.807	2.069	71.299				
11	0.775	1.986	73.285				
12	0.747	1.916	75.201				
13	0.680	1.744	76.945				
14	0.672	1.724	78.669				
15	0.583	1.496	80.165				
16	0.554	1.419	81.584				
17	0.527	1.351	82.935				
18	0.491	1.260	84.195				
19	0.452	1.160	85.355				
20	0.447	1.146	86.501				
21	0.437	1.121	87.622				
22	0.407	1.043	88.665				
23	0.401	1.028	89.694				
24	0.371	0.952	90.645				
25	0.361	0.925	91.570				
26	0.330	0.846	92.416				
27	0.321	0.823	93.239				
28	0.298	0.763	94.002				
29	0.286	0.734	94.736				
30	0.276	0.706	95.443				
31	0.263	0.675	96.118				
32	0.251	0.643	96.761				
33	0.211	0.540	97.302				
34	0.206	0.527	97.829				
35	0.192	0.492	98.321				
36	0.177	0.453	98.774				
37	0.171	0.439	99.213				
38	0.159	0.408	99.621				
39	0.148	0.379	100.000				

Extraction method: Principal component analysis. A. When components are correlated, sums of squared loadings cannot be added to obtain a total variance.

**TABLE 4:** Structure matrix regarding hidden curriculum.

Items	Components					
	1	2	3	4	5	6
I have a good relationship with my teachers.	0.378				−0.742	
The teachers and staff of our institute are highly supportive.	0.457	0.414	0.309		−0.754	−0.340
The confidentiality of our personal and academic records is respected and maintained.	0.405	0.451			−0.711	
The vision of our institutes is progressive.	0.625	0.455			−0.756	
There is open access to all relevant information	0.454	0.325	0.354	0.395	−0.567	−0.382
Teachers, students, and staff work in close collaboration.	0.589	0.330	0.378		−0.733	−0.314
The teacher’s attitude encourages students to ask questions.	0.379	0.562		0.346	−0.527	−0.462
**Teachers concede when they do not know the answers.**		0.341		**0.241**		
Teachers marking is fair and objective.	0.324	0.617	0.325	0.445	−0.457	−0.385
**Teachers share a blueprint of the assessment before the exam.**				**0.287**		
Teachers do not show bias toward selected students.	0.480	0.711		0.378	−0.362	
All students have equal opportunity to participate and excel in academic and non-academic activities irrespective of gender and culture.	0.446	0.772	0.371		−0.512	
Teachers inform students about any change of plan regarding assignments on tests, well in advance.	0.464	0.665	0.325	0.365	−0.474	−0.341
Teachers allow students to explain their points of view without judging them.	0.538	0.692	0.303		−0.380	−0.309
Teachers treat us equally.	0.480	0.819	0.338		−0.521	
There is no gender disparity in our medical institute.	0.584	0.783	0.315		−0.524	
Cultural diversity in our institutes is highly respected.	0.457	0.618	0.453		−0.646	
Teachers and administration respect individuals and value their differences.	0.621	0.727	0.433		−0.641	
Co-curricular activities at our institute foster a healthy and friendly environment.	0.500	0.476	0.673		−0.499	
Our medical institute takes active measures to provide admission to a diverse candidate pool.	0.556	0.542	0.544		−0.543	
Our institute’s policies and procedures encourage diversity, equity, and inclusion.	0.691	0.607	0.401		−0.669	
I believe the administration will take appropriate action in response to incidents of discrimination or bias.	0.698	0.546			−0.513	
Our institute gives us equal and open opportunities to participate in various physical activities.	0.476	0.400	0.767		−0.393	
Our extracurricular activities are highly organized and popular among students.	0.396		0.882			
Our institute provides support and empowerment to the students in all their academic and non-academic growth.	0.658	0.479	0.675		−0.491	−0.313
I am empowered to make important decisions regarding my learning.	0.688	0.644	0.529		−0.517	
I am empowered to make my own choices to achieve my goal, I believe that I am capable of achieving my goals at this institute.	0.799	0.460	0.406		−0.425	
**My association with this institute will help me to do well in the future.**	**0.738**	**0.317**	**0.339**		**−0.336**	
Academic achievement is highly rewarded and recognized in this institute.	0.636		0.568		−0.312	−0.339
I am highly motivated to do well in this institute because I have the power to make difference in how things are done in my class.	0.821	0.380	0.384		−0.451	
Alternative approaches to learning are encouraged in my institutes.	0.798	0.439	0.356		−0.550	−0.459
Learning activities in my institute are intellectually challenging.	0.660	0.391	0.334		−0.473	−0.405
Our curriculum reforms are in line with best international practices.	0.752	0.462	0.385		−0.590	−0.353
Administrative functioning is organized.	0.719				−0.536	−0.362
We are made aware of progressive curricula and driven to explore better opportunities internationally.	0.759	0.370	0.420		−0.470	−0.411
Classroom practices are facilitated by experienced and skilled faculty.	0.516	0.441	0.339		−0.655	−0.632
**Our educational system encourages evidence-based learning.**	**0.663**	**0.340**	**0.323**		**−0.303**	**−0.338**
**Our teachers encourage research and involve students in research activities.**	**0.490**		**0.291**		**−0.300**	**−0.229**
**Academic challenges are easily handled by evidence-based learning.**		**0.233**				**−0.264**

Extraction method: Principal component analysis. Rotation method: Oblimin with Kaiser normalization.

**TABLE 5:** Standardized factors loadings of EFA.

Items	Components		
	CR + RI	SCE	AFS + EO
I have a good relationship with my teachers.	−0.742		
The teachers and staff of our institute are highly supportive.	−0.754		
The confidentiality of our personal and academic records is respected and maintained.	−0.711		
The vision of our institutes is progressive.	−0.756		
There is open access to all relevant information.	−0.567		
Teachers, students, and staff work in close collaboration.	−0.733		
Cultural diversity in our institutes is highly respected.	−0.644		
Our institute’s policies and procedures encourage diversity, equity, and inclusion.	−0.669		
I am highly motivated to do well in this institute because I have the power to make difference in how things are done in my class.	−0.451		
Alternative approaches to learning are encouraged in my institutes.	−0.459		
Learning activities in my institute are intellectually challenging.	−0.473		
Our curriculum reforms are in line with best international practices.	−0.590		
Administrative functioning is organized.	−0.536		
We are made aware of progressive curricula and driven to explore better opportunities internationally.	−0.471		
Co-curricular activities at our institute foster a healthy and friendly environment.		0.673	
Our medical institute takes active measures to provide admission to a diverse candidate pool.		0.544	
Our institute gives us equal and open opportunities to participate in various physical activities.		0.767	
Our extracurricular activities are highly organized and popular among students.		0.882	
Our institute provides support and empowerment to the students in all their academic and non-academic growth.		0.675	
My association with this institute will help me to do well in the future.		0.439	
Academic achievement is highly rewarded and recognized in this institute.		0.568	
The teacher’s attitude encourages students to ask questions.			0.562
Teachers marking is fair and objective.			0.617
Teachers do not show bias toward selected students.			0.711
All students have equal opportunity to participate and excel in academic and non-academic activities irrespective of gender and culture.			0.772
Teachers inform students about any change of plan regarding assignments on the test, well in advance.			0.665
Teachers allow students to explain their points of view without judging them.			0.692
Teachers treat us equally.			0.819
There is no gender disparity in our medical institute.			0.783
Teachers and administration respect individuals and value their differences.			0.727
I believe the administration will take appropriate action in response to incidents of discrimination or bias.			0.546
I am empowered to make important decisions regarding my learning.			0.644
I am empowered to make my own choices to achieve my goal, I believe that I am capable of achieving my goals at this institute.			0.460

CR, *communication and relationship with stakeholders; AFS, accountability of faculty and students in curricular and extracurricular activities; EO, equal opportunity; SCE, student centredness and empowerment; and RI, Evidence based reforms and implementations.

As Rauf et al. (1) extracted five themes by qualitative analysis as communication and relationship with stakeholders (CR), Accountability of faculty and students in curricular and extracurricular activities (AFS), Equal opportunity (EO), Student centredness and empowerment (SCE) and Evidence-based reforms and implementations (RI) while in her continuation of study, these five themes merged into three main factors of the hidden curriculum. The items measure the same underlying construct. The extraction of three factors in the EFA seems to be the result of the wording of the questionnaire items. As a rule of thumb, 10 respondents for one scale item should be selected for an adequate sample size. This study comprized of 301 respondents for 39 items during EFA; hence the model might be underestimated. To check this likelihood, we examined the sample size adequacy using the KMO statistic, and the results showed that 301 respondents were adequate for EFA analysis since the KMO value was greater than 0.7 (18). In many factor analysis investigations, the researcher has some prior information on the communality of the variables and the number of factors present in the study domain based on past research (20). This information can be used to direct the choice of variables to produce a battery with the highest level of communality and the desired amount of overdetermination of the components. Based on our findings, it is preferable for communalities to not fluctuate widely and for the mean degree of communality to be at least 0.7, ideally greater. The idea of content validity, which holds that it is preferable to have more variables (or items) to appropriately describe the domain of each latent variable, is consistent with this notion. The important thing to remember is that these indications must be dependable and reasonably valid. The recovery of population factors will be hurt by the addition of new indicators with low communalities, just as content validity may be harmed by the introduction of additional items that are not representative of the domain of interest. Therefore, adding more indicators for each aspect is only advantageous if the new indicators are accurate assessments of the factors (20). Hence, accountability of faculty and students in curricula and extracurriculars combined with equal opportunity is one factor; communication and relationship with stakeholders combined with evidence-based reforms and implementations is the second factor, and student-centeredness and empowerment are considered the third main factor of the hidden curriculum in medical institutes (1). Further research is warranted to support the questionnaire’s reliability and validity and reproduce the factors that determined the Hidden curriculum with a larger sample to generalize the study’s findings.

## Conclusion

The developed 33-item instrument was effective and reliable to assess the hidden curriculum in medical institutes and hence, accountability of faculty and students in curricula and extracurriculars combined with equal opportunity as one factor; communication and relationship with stakeholders combined with evidence-based reforms and implementations as the second factor, and student-centeredness and empowerment are considered the third main factor of the hidden curriculum in medical institutes. All these three main constructs were collectively used to measure hidden curricula in medical institutes. The study is a work in progress. In the first phase, we worked on identifying themes related to the hidden curriculum (1), next was to develop a validated questionnaire that can measure the construct. The third phase will be to implement the tool and collect data to measure different constructs of hidden curriculum. This will be the “how-to-do” element of the study. The topics and phenomena covered in this article are applicable to confirmatory factor analysis even if the methods utilized in our study were exploratory. Both methods make use of the same factor analysis model that is taken into account in our theoretical framework, and we anticipate that sampling error will have a similar impact on results in confirmatory factor analysis as it did in exploratory factor analysis. The latter may frequently be characterized by indicators with higher commonalities because indicators in such studies are frequently chosen on the basis of established quality as measures of constructs of interest. This is an important distinction between typical exploratory and confirmatory studies. As a result, confirmatory studies may find that the effects of sampling error discussed in this article are less significant.

## Data availability statement

The original contributions presented in this study are included in the article/supplementary material, further inquiries can be directed to the corresponding author.

## Ethics statement

Ethical review and approval was not required for this study on human participants in accordance with the local legislation and institutional requirements. Written informed consent from the patients/participants or patients/participants legal guardian/next of kin was not required to participate in this study in accordance with the national legislation and the institutional requirements.

## Author contributions

AR and FF conceptualized and designed the study. NS performed the data analysis and interpret the results. RG critical reviewed and helped in writing and editing. All authors contributed to the article and approved the submitted version.
